# Diffusion and perfusion weighted magnetic resonance imaging for tumor volume definition in radiotherapy of brain tumors

**DOI:** 10.1186/s13014-016-0702-y

**Published:** 2016-09-21

**Authors:** Lu Guo, Gang Wang, Yuanming Feng, Tonggang Yu, Yu Guo, Xu Bai, Zhaoxiang Ye

**Affiliations:** 1 Department of Biomedical Engineering, Tianjin University, Tianjin, 300072 China; 2 Department of Radiation Oncology, Tianjin Medical University Cancer Institute & Hospital, Tianjin, 300060 China; 3 Department of Radiation Oncology, East Carolina University, 600 Moye Blvd, Greenville, NC 27834 USA; 4 Department of Radiology, Huashan hospital, Fudan University, Shanghai, 200040 China; 5 Department of Radiology, Tianjin Medical University Cancer Institute & Hospital, Tianjin, 300060 China

**Keywords:** Brain tumors, Diffusion, Perfusion, Radiotherapy, Tumor volume definition

## Abstract

Accurate target volume delineation is crucial for the radiotherapy of tumors. Diffusion and perfusion magnetic resonance imaging (MRI) can provide functional information about brain tumors, and they are able to detect tumor volume and physiological changes beyond the lesions shown on conventional MRI. This review examines recent studies that utilized diffusion and perfusion MRI for tumor volume definition in radiotherapy of brain tumors, and it presents the opportunities and challenges in the integration of multimodal functional MRI into clinical practice. The results indicate that specialized and robust post-processing algorithms and tools are needed for the precise alignment of targets on the images, and comprehensive validations with more clinical data are important for the improvement of the correlation between histopathologic results and MRI parameter images.

## Introduction

### Background

#### Brain tumors

According to the World Health Organization (WHO) classification, high-grade gliomas are the most malignant primary intra-axial brain tumors in adults, and most cases consist of glioblastoma multiforme (GBM, WHO grade IV astrocytoma). Low-grade gliomas are much less common, and most are WHO grade II gliomas [1]. Meningiomas are the most common primary extra-axial brain tumors, and they account for approximately 20 % of all brain tumors [2, 3]. Further, malignant and atypical meningiomas are more prone to recurrence and aggressive growth, increasing patient morbidity and mortality [4]. Brain metastases are the second most common intracranial tumors in adults (15–30 % of all intracranial tumors) [5]. Most of them originate from lung cancer (16–20 % incidence), melanoma (7 % incidence), renal cell carcinoma (7–10 % incidence), and breast cancer (5 % incidence) [6]. Therefore, we focus on these brain carcinomas in this review.

#### Limitations of conventional magnetic resonance imaging (MRI) in radiotherapy for brain tumors

Radiotherapy plays an important role in the treatment of brain tumors [7]. To avoid missing the target and to spare critical healthy brain tissue outside the target volume in radiotherapy, cancerous tissue involvement must be correctly defined in the gross tumor volume (GTV) delineation. Patients undergo a contrast-enhanced computed tomography (CT) scan in the treatment position as an initial step in radiotherapy planning for the delineation of target volumes and organs at risk (OAR), as well as treatment beam setup and dose calculation [7]. However, there are inherent limitations to CT in showing satisfactory contrast in soft tissue, and it is insufficient for obtaining comprehensive information about tumor and the desired accuracy of the delineation. As a complementary technique, conventional T1-weighted contrast-enhanced and T2-weighted MRI reveal soft tissues with higher contrast and could help improve the accuracy of tumor volume definition [7, 8]. The significant practical value of the integration of MR/CT data into treatment planning in reducing target volumes and interobserver variability has been proved [9–11]. Furthermore, MRI-based treatment planning using MRI simulation for intracranial lesions has been developed and has shown no significant differences compared with CT + MRI plans in tumor control probability [12]. Recently, combined positron emission tomography (PET)/MRI has become available in clinical practice, offering the possibility of imaging the anatomical and functional characteristics of a tumor simultaneously, and it could improve radiotherapy treatment planning and the evaluation of treatment response [13, 14].

Although conventional MRI has been used in tumor volume definition for nearly 20 years, it does not accurately show the actual tumor borders of glial neoplasms because tumor cells could be found in serial biopsies beyond signal intensity changes on T2‑weighted MRI images [15]. Because one of the major factors in treatment failure of gliomas is the invasion of tumor cells into normal brain tissue, the definition of exact tumor borders has been essential for safe and successful treatment administration [16]. Currently, larger margins (e.g., a 2.0–2.5-cm margin for WHO grade IV glioma) are added to the GTV to generate the clinical target volume (CTV), which aims to cover the microscopically pathologic volume around the apparent tumor, including peritumoral edema and infiltrating cells [1, 17, 18]. However, the irradiated volume defined with this method oftentimes involves normal brain tissue, which is radiosensitive. It limits the total dose that can be delivered to the target volume for killing tumor cells, resulting in tumor recurrence within the target volume [16, 19]. Furthermore, in the retreatment of recurrent tumors, the risk of radiation necrosis and the conservation of normal brain tissue should be considered for the delineation of accurate tumor extent. Unfortunately, conventional MRI has shown significant limitations, not only in tumor detection, target delineation and differentiation of the tumor from other disease processes before radiotherapy but also in the discrimination of radiation treatment effects and recurrent tumors after radiotherapy [20–22].

#### The potential value of advanced methods of diffusion and perfusion MRI

The development of advanced imaging techniques, such as diffusion and perfusion MRI, has enabled more sensitive detection of the heterogeneity and invasion of tumors than with conventional MRI. These novel methods provide functional information about tumor cellularity, proliferation, disruption of white matter, tumor vascularity and vessel permeability. All of these physiological changes are involved in the tumor progression and invasion processes, which could allow for improved tumor delineation and assessment of tumor extent in radiotherapy for brain tumor patients.

### Objectives

The purpose of this review is to examine recent studies that have included the utilities of diffusion and perfusion MRI for tumor volume definition during the course of radiotherapy for brain tumors, including treatment planning and therapeutic response assessment. Both opportunities and challenges for the integration of these advanced functional imaging techniques into clinical practice are presented.

## Review

### Diffusion MRI

#### Diffusion features and metrics

Diffusion-weighted imaging (DWI) measures random water movement in tissue, and its derived parameter of apparent diffusion coefficient (ADC) represents direction-independent water displacement. In tissues with high cellularity, free water motion is restricted, and the measured ADC is low, whereas in tissues with low cellularity, the corresponding ADC is high [3]. This inverse correlation between ADC and cellularity may serve as a tool to differentiate more and less malignant glial tumors. For meningiomas, the ADC map could be used to differentiate the solid tumor from edema because the ADC value in edematous areas is significantly higher than that in solid tumor and normal brain tissue. Moreover, ADC has recently been shown to be correlated with survival for brain metastases [23].

In diffusion tensor imaging (DTI), diffusion-weighted images are acquired with six or more gradient directions, and three eigenvalues of the diffusion tensor are calculated (λ_1_, λ_2_, λ_3_) to evaluate the water molecules’ movement on the X, Y and Z axes [24, 25]. Based on the three eigenvalues, different tensor metrics, such as fractional anisotropy (FA) and mean diffusivity index (MD), can be calculated to quantify the amount of directional water motion. The FA value ranges from 0 to 1, depending on the degree of directionally ordered brain tissues in which water motion occurs, especially the brain fiber alignment. For instance, 0 indicates that the water molecules move in a completely homogeneous medium such as cerebrospinal fluid (CSF), and one indicates water movement in well-organized and highly directional structures, such as the axons and myelin sheathes of white matter. Thus, the FA value is sensitive to the changes in fiber bundles and might allow for better evaluation of the disruption, displacement and infiltration of white matter caused by tumor invasion in regions that appear normal on conventional MR images. Furthermore, DTI fiber tractography, which is calculated based on a specific sub-voxel fiber tracking algorithm, can depict how the presence of a brain tumor has disrupted the fiber integrity [24, 26]. Because gliomas preferentially infiltrate along white matter tracks, DTI could be used to predict the relapse patterns of tumors in radiation treatment planning [27].

#### Diffusion MR in treatment planning

The purpose of an MRI exam for radiotherapy treatment planning is to utilize image information to create a precise delineation of the target volume and to obtain an optimal dose distribution [28]. Advanced imaging techniques might be more promising for assessing the microenvironment within a tumor, such as the cellular density, organization of the cellular and extracellular space, margin of tumor invasion, and white matter involvement in tumor infiltration, compared with conventional anatomical MR images. Diffusion metrics and fiber tractography derived from DWI/DTI imaging techniques have been investigated to define accurate tumor extent and delineate target volume in radiation treatment planning and to guide surgery/biopsy sampling [26, 29–38].

For high-grade gliomas, standard CT and MRI are unable to demonstrate subclinical tumor spread, resulting in insufficient doses to peripheral regions of the tumor to achieve good local tumor control. A new term for such abnormalities, image-based high-risk volume (IHV), which is distinct from GTV, was proposed by Jena et al. for assessment of the value of DTI in the individualization of radiotherapy treatment planning for patients with high-grade gliomas [29]. In their study, the non-uniform IHV was added to the GTV (defined on CT or MRI) to include areas of abnormality on DTI, and the CTV was generated by adding a 1-cm margin to the IHV. Compared with the normal standard plan, the size of the DTI-based planning target volume (PTV) was reduced by approximately 18 to 46 % (mean 35 %), and it encompassed areas at high risk of tumor involvement and resulted in escalated doses without an increase in normal tissue complication probability (NTCP). In a recent report, the isotropic component (*p*) and anisotropic component (*q*) of the water diffusion tensor, calculated by DTI data, were used to extend the CTV along the abnormal tensor tracts to preserve coverage of the likely routes of dissemination, while sparing uninvolved brain tissues [30]. Thus, although different areas within the standard CTV have different probabilities of tumor infiltration, DTI seems to show that a higher risk of infiltration occurs around the tumor center, and it can be used as the basis for the individualization of treatment volumes for high-grade glioma patients [29].

Several novel methods have been investigated with DTI metrics to improve the definition of tumor infiltration margins in radiotherapy for malignant glioma patients. As proved in a study of 17 patients with surgically treated WHO grade IV gliomas, the ADC and FA values could be used to identify ultra-structural changes in perilesional hyperintensity areas (FA was significantly lower and ADC significantly higher than in the unaffected contralateral tissue) and to help determine the tissue at risk of tumor infiltration [32]. Price et al. showed that it was possible to use the anisotropic component (*q*) and isotropic component (*p*) of the water diffusion tensor to differentiate gross tumor (decrease in *q* was more than 12 % than that in the contralateral region) from tumor infiltration (increase of *p* was more than 10 % than that in the contralateral region), which could serve for better delineation of the tumor margin for glioma treatment [33]. Moreover, a new quantification method to solve problems in the reproducible determination of DTI-derived FA values was reported and showed its feasibility for the reliable depiction of white matter infiltration by gliomas [39]. Thus, when the tumor is near critical, sensitive structures, and a stricter boundary definition is required for radiation treatment, DTI could serve as a promising tool to identify tissue at risk of tumor infiltration and to spare uncancerous brain tissue [32].

Based on the knowledge that glioma cells preferentially spread along nerve fibers, there are new methods to estimate the optimal radiation target volume to encompass the whole tumor. The computational simulation model for the pattern of glioma spread with white fiber distribution information, based on DTI data, might be one of the most promising approaches for estimating tumor growth and determining the tumor margin for radiotherapy, and it can help deliver a radio-surgical boost to “high-risk” volumes in which the likelihood of finding malignant cells is high [40]. Another method for identifying the tumor invasion margin correctly was proposed that uses a geodesic distance on the Riemannian manifold of brain fibers to replace the Euclidean distance used in clinical practice [31]. In this study, Cobzas et al. used actual DTI data from patients with gliomas to compute the geodesic distance, and they compared their predicted tumor growth with follow-up MRI scans. Compared to the conventional Euclidean distance, the geodesic distance method could significantly improve the prediction of glioma invasion margins, and these computational simulation methods for the prediction of the invasive margin of glioma showed potential for the delineation of optimal radiation target volumes, although further studies with more patient data are necessary to validate their feasibility in clinical practice.

With DTI-derived fiber tractography, white matter involvement by a tumor can be classified as displacement, infiltration or disruption, compared with the normal unaffected tract on the contralateral side [36]. Fiber tractography is critical not only for defining the effects of invasive gliomas on white matter tracts but also for demonstrating the relationships between white matter structures and the cerebral neoplasm. Currently, this technique has been employed in academic research to guide surgical or radiation treatment planning to preserve vital tracts while maximizing tumor resection or local control [26, 35–37]. However, there currently are several obstacles to integrating tractography into clinical treatment planning systems. First, there is no “gold standard” for accurately validating the reliability of in vivo tractography [26]. Second, image-derived results cannot yet determine the actual size of white matter tracts [37]. With the continuous improvement of this technology, however, fiber tractography still holds great promise for the definition of cerebral tumor invasion into white matter and for the improvement of radiotherapy treatment.

#### Diffusion MR in the assessment of treatment response

For noninvasive tumors, such as meningiomas and metastases, the conventional method to assess treatment response is by changes in tumor volume on contrast-enhanced T1-weighted MRI, while it is difficult to differentiate therapy-related effects from tumor recurrence in high-grade gliomas or anaplastic transformation in low grade gliomas with conventional MRI [3]. With abnormal hypercellularity and microvasculature, the invasive growth of high-grade gliomas results in breakdown of the blood-brain barrier (BBB), shown as a volume of contrast enhancement on T1-weighted MRI images. The edema and infiltration surrounding the tumor without BBB disruption appear as hyperintensity on T2-weighted MRI images but non-enhancement on T1-weighted MRI images. Low-grade gliomas typically exhibit no contrast enhancement, but they become contrast enhancing when there is anaplastic transformation to high grade. However, the appearance becomes complex after therapy because the treatment-induced effects, such as necrosis, also lead to BBB breakdown, similar to tumor progression and recurrence. Recent studies have investigated advanced imaging methods, such as DWI and DTI, for the reliable identification of these entities and the adjustment of radiotherapy planning as early as possible.

A study of DTI-derived ADC values in new contrast-enhancing lesions and perilesional edema in 28 patients previously treated for brain tumors (GBMs, oligodendrogliomas and astrocytomas of grades II and III) was reported, and these values showed their capacity to distinguish recurrent neoplasms from treatment-related injuries [41]. Although a high cell-density tumor and radiation-induced injury both restrict water mobility and lead to a lower ADC, the increased extracellular space and micronecrosis in areas of tumor recurrence can eventually cause higher ADC values [41]. Thus, ADC values in contrast-enhancing lesions and ADC ratios (ADC in the pathological area divided by ADC in the mirror contralateral area) in the white-matter tracts in perilesional edema were significantly higher for the recurrence group than for the non-recurrence group (*P* = 0.01). Additionally, FA values in contrast-enhanced lesions after radiotherapy for patients with astrocytomas may also be used to differentiate radiation necrosis from tumor recurrence [41, 42]. Although astrocytomas tend to destroy the integration of white matter fibers and cell structures, resulting in decreased FA values, histological characteristics such as cellularity and vascularity also influence FA values, finally causing tumors of higher grade to show higher FA values in contrast-enhanced areas. In contrast, radiation necrotic tissue with no normal fibers accounts for the lower FA value in the enhanced lesion than that of the recurrent tumor [42], making it possible to differentiate radiation necrosis from tumor recurrence using FA values.

For the early prediction of response to the treatment of gliomas, Lemasson et al. evaluated a voxel-based image biomarker, the functional diffusion map (fDM), to detect the heterogeneity of tumor response in a radiation dose escalation protocol [43]. By first calculating the difference between the ADCs before and after therapy, fDM was determined within the total tumor and was classified into three categories -- fDM+, fDM− and fDM0 -- indicating increased, decreased, and unchanged ADC, respectively. The study showed that treatment induced a dose-dependent increase in ADC values of tumors, and fDM+ was able to improve the detection of dose-dependent trends in tumor response. Thus, fDM could be used in the future for adaptive conformal targeting of specific tumor regions exhibiting resistance [43].

In the assessment of radiation-induced white matter injury, DTI is regarded as the most sensitive technique for the identification of white matter abnormalities before anatomical changes on conventional MRI images. A recent analysis showed that ADC values based on DTI could serve as a biomarker for the determination of long-term radiation-related injury to white matter [44]. The results demonstrated that the structural integrity of white matter underwent changes after radiation therapy starting as early as when the received total dose reached 5 to 15 Gy. Furthermore, a mean ADC increase of 8 %, compared to normal white-matter tissue, was detectable at total radiation dose levels of 15 to 45 Gy because of diffuse post-irradiation white matter injury, such as reactive astrocytosis, edema or demyelination. Thus, this technique has shown great promise in radiation-induced white-matter injury assessment, but rigorous clinical validation is also required to evaluate its potential in treatment response assessment.

For the management of GBM patients with recurrence after primary radiotherapy, re-irradiation is one of the salvage treatment options. However, treatment-related morbidity becomes a major concern due to the reduced radiation tolerance of normal tissue after initial treatment [45]. Thus, the improvement of techniques for target definition and radiation dose delivery plays a critical role in safe radiation administration with fewer adverse effects. The retreatment target volume should therefore be minimized to decrease the probability of normal brain necrosis and to improve the overall survival of recurrent GBM patients [46, 47]. Using the tissue signature method, Price et al. defined a region around a tumor with a reduced anisotropic component (*q*) due to tumor disruption and an area outside the increased isotropic component (*p*) due to tumor infiltration [48]. This technique could lead to smaller treatment volumes by tailoring the high-dose region to high-risk areas for subclinical spread [49]. Furthermore, it has been shown that 95 % of tumor progression occurs within 2–3 cm of the resection cavity, and the vast majority of patients (>80 %) continue to recur within 2 cm of the re-irradiated volume [45, 50]. Thus, Krishnan et al. determined the direct and unique clinical utility of DTI tractography in treatment planning, in which target volumes were modified to provide elongated treatment margins along the paths of elevated water diffusion, creating biologically better treatment that may reduce the incidence of progression [50]. Therefore, DTI has high inherent ability to improve radiotherapy target delineation by creating a biologically better treatment plan that may reduce the incidence of progression [51].

The utilization of DWI/DTI metrics is summarized in Table 1. These studies revealed that diffusion MRI is critical for making decisions about tumor delineation with more accurate margins in treatment planning. The factors that influence these DTI metrics include edema, tumor necrosis, the density of tumor cells, and treatment effects. Thus, further validations with detailed histologic sampling improved the placement of regions of interest (ROIs), and reduced errors in multimodal image registration are necessary for the application of these parameters to obtain valuable information for tumor volume definition in radiotherapy.

**Table 1:** The utilization of DWI/DTI-derived metrics in brain tumor treatment course

Utilities	DWI	DTI					
	ADC	MD	FA	ADC	Eigenvalues	Tractography	Isotropic (*p*) and anisotropic (*q*) components
Tumor cellularity	[75]	[38]	[38]				
Prognosis							[34]
Invasion			[31, 32, 39]	[32]			[33]
RT treatment planning			[29, 30]				[30, 33]
Surgery guidance		[38]	[36–38]	[36]		[26, 35]	
Response to treatment	[43]						
Treatment effect				[44]			
Distinguishing tumor recurrence from treatment effect			[41, 42]	[41]	[41]		
Re-irradiation treatment planning						[50]	[48]

*DWI* diffusion weighted imaging, *DTI* diffusion tensor imaging, *ADC* apparent diffusion coefficient, *MD* mean diffusivity, *FA* fractional anisotropy, *RT* radiotherapy

### Perfusion MRI

#### Perfusion MRI and vascular properties

Malignant gliomas are able to recruit and synthesize vascular networks for growth, proliferation and metastasis. Because of rapid tumor cell growth, the vasculature of malignant gliomas may consist of a high density of immature and leaky blood vessels in the periphery of the tumor and regression and low density of vessels in the core of the tumor [19]. Without new blood vessels that form to supply oxygen and nutrients to cells, the diameter of the solid tumor volume can only reach 2 mm. Thus, vascularity is crucial to the categorization of the malignant potential of intra-axial tumors, with direct consequences for therapeutic management [3], whereas in benign neoplasms such as meningioma, vascular proliferation occurs by recruiting existing capillaries and not angiogenesis so that biopsies of high vascularity are not observed in these tumors [16, 52]. Several advanced MRI techniques have been investigated to assess the vasculature of brain tumors with perfusion evaluation, including dynamic-susceptibility contrast (DSC), dynamic-contrast enhanced (DCE) and arterial spin-labeling (ASL) techniques. For DSC and DCE MRI, intravenous injection of a bolus of gadolinium-diethylenetriaminepentaacetic acid (Gd-DTPA) is necessary to assess parameters such as cerebral blood volume (CBV), cerebral blood flow (CBF) and transfer constant (K^trans^).

DSC imaging uses a series of T2*‑weighted echo planar MR sequences to assess the first-pass of a contrast bolus in brain tissue in which signal loss occurs because of the increase in contrast agent concentration. According to the indicator dilution theory for intravascular tracers, voxel-based CBV maps can be created by integrating the area under the concentration-time curve of the contrast agent. With the measurement of arterial input function (AIF) and pharmacokinetic models, the CBF map can be estimated voxel by voxel. Another common parameter map that depicts vascular properties is the mean transit time (MTT), which can be easily calculated according to the central volume theorem as follows [53]:

1 $$ CBF=\frac{CBV}{MTT}. $$

**Fig. 1:**
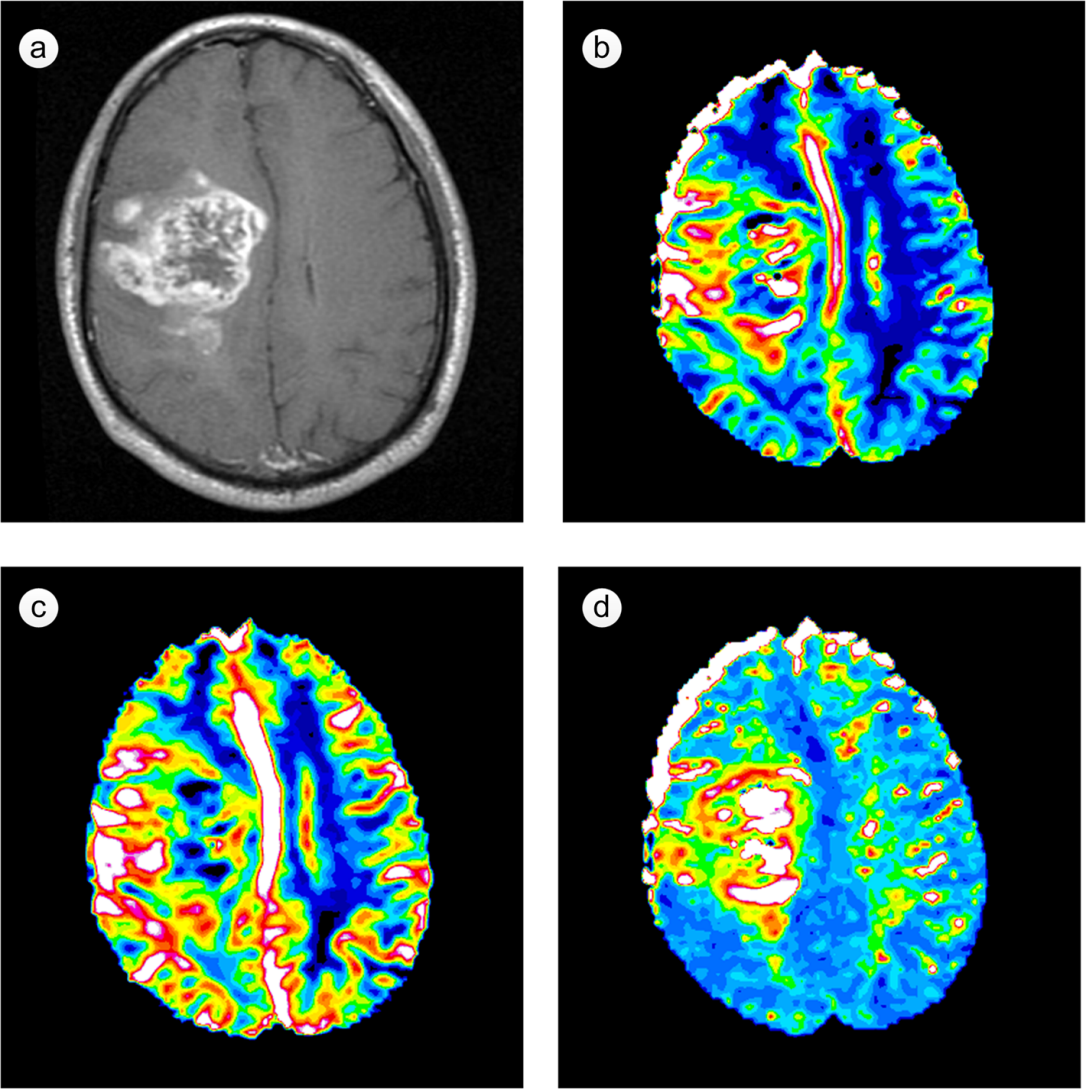
Images of a patient with an untreated glioblastoma multiforme (GBM). **a** T1-weighted contrast-enhanced image; **b** relative cerebral blood volume (rCBV) map; **c** relative cerebral blood flow (rCBF) map; **d** relative mean transit time (rMTT) map

In clinical practice, these quantities are often not calculated for absolute but for relative measurement, and they are called relative CBV (rCBV), relative CBF (rCBF) and relative MTT (rMTT). Additionally, several time-related parameter maps, such as the percentage of base at peak (PBP) and time to peak (TTP), are measured for the detection of abnormal signals in the brain tumor tissue. The overall tumor vascularity can be depicted by these parameter maps derived from perfusion MRI, such as rCBV, rCBF and rMTT (Fig. 1), and the local variations in regional parameters could reflect the tumor vascularity alterations, which are correlated with tumor angiogenesis [54].

DCE imaging uses T1‑weighted sequences to identify the changes in the MR signal when the bolus of contrast agent passes through the blood vessels and leaks into the interstitial space as the BBB is disrupted. Therefore, dynamically generated images can reveal regional microvascular properties, such as vascular permeability (which can be depicted by K^trans^) and the extravascular extracellular space (Ve) in and around the tumor. The estimation of parameters derived from the dynamic data includes K^trans^, fractional blood volume (fBV), and flux rate constant (Kep), which have been found to be correlated with tumor grade, microvascular density (MVD), tumor recurrence and survival outcome [3, 24].

In contrast to DSC and DCE, ASL employs water molecules in tissue as a freely diffusible intrinsic tracer to measure CBF in tumor tissue. Upstream of the imaging section, reversed phase or saturated radiofrequency pulse sequences mark the hydrogen protons in arterial blood. When the magnetically labeled protons transfer from the labeling section into the imaging region through the bloodstream, they interact with the non-labeled tissue water in the capillaries. Thus, the static magnetization vector in tissue decreases because of blood flow and T1 relaxation, resulting in regional changes in signal intensity. Subsequently, rCBF maps are calculated by the comparison of the T1 relaxation time between images acquired with and without magnetically labeling. As a totally noninvasive and quantitative technique, ASL has shown promise in prognosis and treatment response evaluation for brain tumors.

#### Perfusion MRI in tumor volume definition

Accurate margin definition for gliomas has been a challenge in radiotherapy. The tumor perfusion parameter maps derived from DSC and DCE imaging, including rCBV, rMTT and K^trans^, provide information about tumor vascularity that is reflective of tumor invasion and allows for improved tumor volume delineation [55–58], as well as determination of aggressive or infiltrating tumor sub-volume in radiotherapy treatment planning [59, 60].

The significant tissue heterogeneity in gliomas, consisting of varying amounts of cellular and nuclear pleomorphism, mitotic activity, microvascular proliferation and necrosis, leads to the signal diversity in perfusion imaging data [54]. Thus, isolated perfusion parameter is insufficient, and multiparametric methods should be developed for complementary depiction of the whole tumor, which could result in better definition of tumor margins. Durst et al. applied a multimodal imaging scheme with rCBV, rCBF, rMTT, K^trans^, MD and FA to predict the extent of glioma invasion with a significant correlation with histopathology [59]. In their study, advanced multimodal neuroimaging sequences and stereotactic biopsy coordinates were co-registered, and the perfusion and diffusion parameters were assigned to the biopsy sites. Multivariate analysis was used to build the final model describing nuclear density, and it showed apparent correlation between the predicted and observed nuclear density. Therefore, with the multiparametric scheme, the radiation oncologist could shape the radiation field more accurately, treat the sites most likely to recur while sparing healthy tissue, and ultimately improve patient outcomes [59].

There are different components in a glial neoplasm, including cancerous tissue, pure edema, cellular proliferation, vascular proliferation, cellular infiltration and necrosis, and the number of components increases with the tumor grade. To differentiate different biological tumor components and to determine tumor borders between peritumoral edema and infiltration areas, rCBV values in different morphological areas (e.g., solid appearing, edematous appearing) were evaluated [55]. In the perienhancing areas with abnormal signals on conventional contrast-enhanced MR images for gliomas, rCBV proved significantly higher in cancerous tissue, lower in normal tissue, and intermediate in the infiltrating tumor, perhaps providing useful information for improving the definition of glioma extent and grade [56]. For malignant gliomas such as GBMs that exhibit extensive infiltration in the surrounding tissue along the white matter tracts and perivascular spaces, rCBV shows statistically significant differences in different apparently edematous areas surrounding the enhanced margins of GBMs. The results of calculating the ratio of rCBV in the tumor to rCBV in the contralateral normal white matter in Di Costanzo’s report showed a higher rCBV ratio (mean value = 1.42) in the infiltrating tumor, a lower rCBV ratio (mean value = 0.36) in pure edema, and an intermediate rCBV ratio (mean value = 0.97) in tumor-infiltrated edema [57]. The findings in this study could have significant implications for improving the evaluation of GBM extent in radiation treatment planning. For further practice, clinical trials to correlate multimodal MR data with histopathological findings are needed. Furthermore, rather than determining the tissue to be either healthy or tumorous, considering it as a continuum from healthy tissue to poorly differentiated tumor tissue might be more meaningful [61].

#### Perfusion MRI in therapeutic response assessment

In the assessment of tumor response to treatment, i.e., distinguishing tumor recurrence or progression from treatment effects in brain tumors, the measurements of rCBV, rCBF and K^trans^ in DSC, DCE and ASL have shown advantages beyond conventional MRI.

For high-grade gliomas, especially GBM, monitoring the treatment response to radiotherapy remains difficult because the frequent occurrence of early changes on contrast-enhanced MR images, such as radiation necrosis, can mimic tumor progression, which is called pseudo-progression. Although the precise mechanism of post-radiotherapy pseudo-progression remains unknown, there is a hypothesis that radiation-induced vascular changes may lead to a focal transient increase in gadolinium enhancement [62, 63]. In addition, the tumor-mimicking lesions of pseudo-progression lack angiogenesis and are stabilized without changes during treatment, different from real tumor progression [3]. Thus, the vascular property measurements, such as cerebral blood volume and vascular permeability, are promising for distinguishing true progression from pseudo-progression. An innovative method called parametric response mapping (PRM) was applied to quantify early hemodynamic alterations after treatment by calculating the differences between serial rCBV maps (ΔrCBV = rCBV_intratreatment_ – rCBV_pretreatment_) for each voxel within the GTV [63]. Furthermore, a significantly reduced blood volume (PRM_rCBV_) at week 3 in the progressive disease group compared with the pseudo-progression group (*P* < 0.01) shows the potential ability of rCBV in distinguishing pseudo-progression from true progression in patients with high-grade glioma. Another new imaging biomarker of tumor progression based on the measurement of rCBV, called pMRI-fractional tumor burden (pMRI-FTB), was also proposed, and it determines the percentage of tumor voxels relative to total lesion mask voxels using the optimized rCBV threshold. Moreover, pMRI-FTB is significantly correlated with the histologic tumor fraction (*P* < 0.0001) and overall survival (*P* < 0.02) [64]. Moreover, in a recent report, relative K^trans^ (rK^trans^), initial area under the concentration curve (riAUC) and rCBV value in the recurrent group proved to be significantly higher than the values in the group with treatment-induced changes, and DCE showed no significant differences from DSC in differentiating power [65]. These methods hold great potential for treatment response assessment and could find greater application in clinical practice with more precise and detailed investigation in a larger glioma population.

In addition to gliomas, rCBV measurement in tumor and normal tissue after radiotherapy has been used to evaluate the response of extra-axial brain tumors such as meningiomas. Because the location of a neoplasm is near the skull base and orbit, susceptibility artifacts can lead to image distortion in perfusion-weighted echo planer imaging sequences (EPI) such as DSC. To resolve this problem, a recent study performed pseudo-continuous arterial spin labeling (pcASL) methods and showed that the maximum tumor blood flow (mTBF, the maximum rCBV value in ROIs placed in tumor tissue showing enhancement) was correlated significantly with the changes in tumor volume before and after radiotherapy [66]. According to the results, mTBF could serve as a potential biomarker of tumor volume for assessing therapeutic response without contrast agents, and future investigations should also be conducted by comparing the advantages of pcASL to those of other perfusion imaging techniques such as DSC and DCE.

For prognosis, the alterations of rCBF in ASL and DSC imaging at the 6-week follow-up after stereotactic radiosurgery for brain metastases were highly predictive of treatment outcome [5]. A recent study investigating radiation response for brain metastases also reported that responders showed a significant lower square of K^trans^ (K_trans_^2^) and reduced rCBF at the 1 week follow-up, and progressive lesions showed significantly lower rCBF and reduced rCBV at the 1-month follow-up [67]. Considering that the differences in radiation dose among different patients can cause changes in vascular perfusion and permeability, clinical trials investigating the impact of biologically effective doses on parameter maps are needed for the further validation of perfusion imaging techniques for prognosis.

In the re-irradiation treatment planning of recurrent high-grade gliomas, perfusion MRI is mainly performed for the purpose of differentiating recurrent tumors from radiation changes, along with pseudo-progression as mentioned above. In addition, several investigations have begun to incorporate perfusion MRI into re-irradiation treatment plans for tumor border identification of recurrent brain tumors. For instance, Kim overlaid rCBV and radiation dose maps on conventional MR to delineate differences in identified tumor burdens for patients with recurrent high-grade glioma [68]. According to the results, rCBV maps of one-half of the patients showed tumors outside the planning volumes, while the remainder showed that the treatment volumes were overlaid with uninvolved brain tissues. Thus, rCBV maps proved to be helpful in determining unique regions of potential tumor burden in recurrent high-grade glioma compared to conventional MRI. Moreover, semi-quantitative histogram analysis of the normalized CBV for contrast-enhanced lesions showed potential in the prediction of the volume fraction of post-treatment high-grade glioma recurrence, which could help in the determination of tumor extent [69]. Additionally, the stripe-like pattern of increased rCBV adjacent to the contrast enhancement on T1-weighted MRI of GBM at follow-up might indicate infiltration transforming into future tumor recurrence [70]. The treatment volume could be modified according to the area with increased rCBV to target the high-risk biological marginal recurrence and to reduce exposure of viable brain tissue to high radiation doses.

The utilities of PWI metrics are summarized in Table 2. To define accurate tumor-invasive borders for radiation treatment, rCBV derived from DSC seems to be the most common and promising parameter because it shows significant differences among peritumoral edema, infiltration areas and normal tissue. To become a tool in clinical routine, it would be desirable to conduct further investigations with larger study populations and detailed histopathologic findings and to strengthen the validity of these parameters as imaging markers to define the exact location of the border between tumor and healthy tissue.

**Table 2:** The utilization of PWI-derived metrics in brain tumor treatment course

Utilities	DSC					DCE	ASL
	rCBV	rCBF	rMTT	K^trans^	K^trans^/rK^trans^	riAUC	rCBF
Prognosis	[67]	[5]			[67]		[5]
Invasion	[58]		[59]	[59]			
RT treatment planning	[55–57]						
Response to treatment	[63]						[66]
Progression vs. pseudo-progression	[63]						
Distinguishing tumor recurrence from treatment effect	[64, 65]				[65]	[65]	
Re-irradiation treatment planning	[68–70]						

*DSC* dynamic susceptibility-weighted contrast-enhanced perfusion MRI, *DCE* dynamic contrast-enhanced MRI, *ASL* arterial spin-labeling, *rCBV* relative cerebral blood volume, *rCBF* relative cerebral blood flow, *rMTT* relative mean transit time, *K* ^*trans*^ transfer constant, *rK* ^*trans*^ relative K^trans^, *riAUC* initial area under the concentration curve

### Integration of multimodality imaging data

**Fig. 2:**
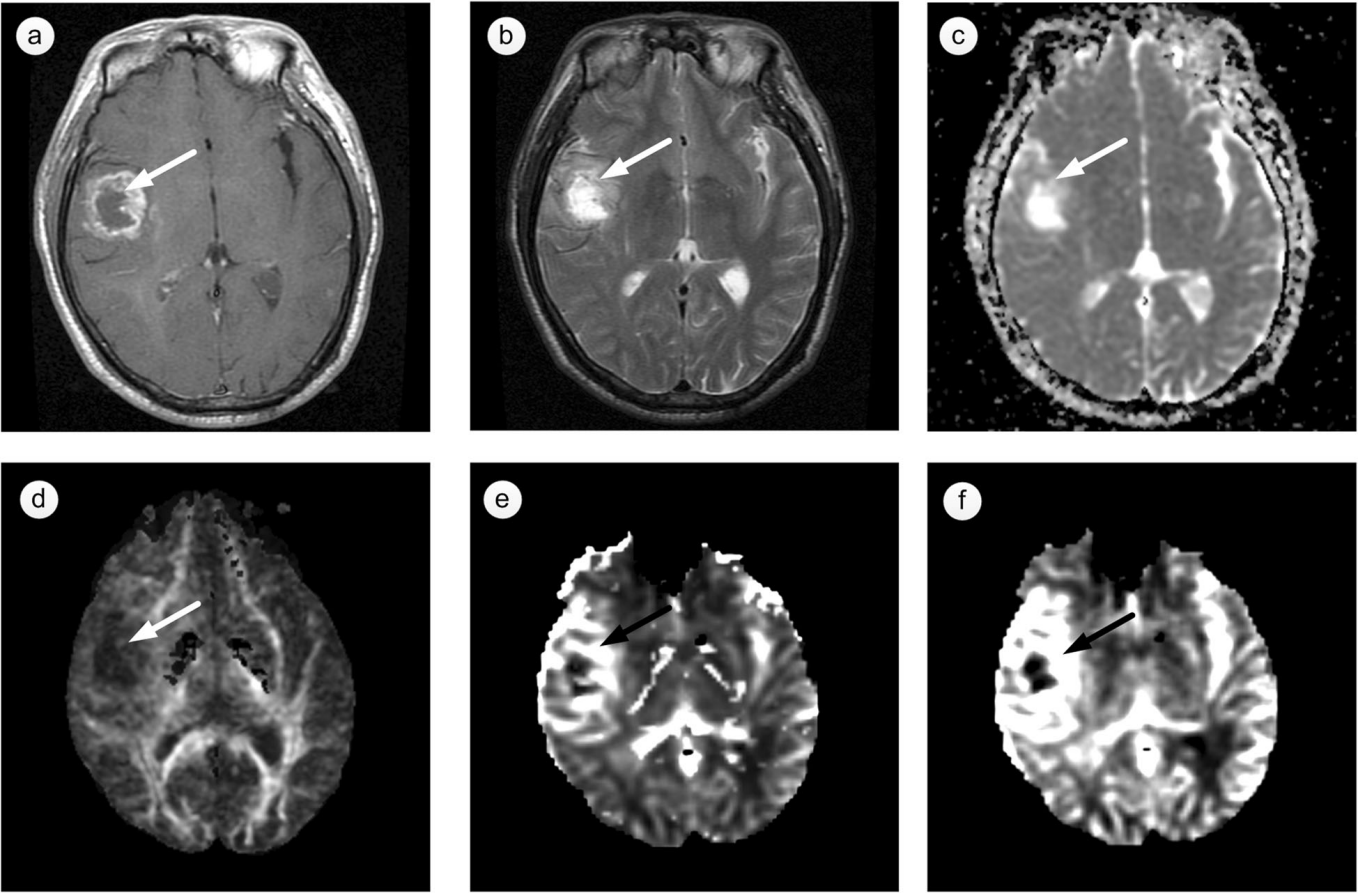
Multimodal images of a patient with glioblastoma multiforme (GBM). **a** T1-weighted contrast-enhanced image, **b** T2-weighted image, **c** apparent diffusion coefficient (ADC) map, **d** fractional anisotropy (FA) map, **e** relative cerebral blood volume (rCBV) map, **f** relative cerebral blood flow (rCBF) map. In the heterogeneous enhancement region (indicated by *arrows*), these properties on anatomical and functional images are different from each other

Isolated diffusion/perfusion parameters only reveal partial information about brain-tumor microenvironments and are insufficient to enable clinicians make crucial decisions throughout the whole treatment. Properties associated with the presence of a tumor might not be correlated with each other perfectly (Fig. 2), and characteristics examined by different imaging techniques do not co-localize well in all patients [61]. For instance, ADC essentially reflects the density of cells and the extra-cellular extra-vascular space, whereas K^trans^ reflects the micro-vessel density and permeability of the vasculature [61]. Thus, the combination of advanced MR imaging techniques such as DWI/DTI, DSC and DCE is able to assess tumor cellularity, infiltration, and vascularity properties simultaneously. The integration of multimodal imaging techniques has been investigated with increasing interest in recent studies and has shown promise in tumor differentiation, grading, accurate tumor extent definition and therapeutic response determination. For example, the integration of parameter maps such as ADC, FA and rCBV derived from diffusion and perfusion MRI data have been used to differentiate among GBMs, solitary brain metastases, and primary cerebral lymphomas [71] to serve as indicators of glioma proliferation [72], to predict tumor infiltration in patients with gliomas [59], to evaluate glioma grading and to segment tumor, edema, necrosis, CSF and normal tissues [73].

Additionally, an imaging technique for the evaluation of tissue metabolism in tumors, called proton magnetic resonance spectroscopic imaging (^1^H-MRSI), has also been integrated into the multiparametric MRI assessment of the accurate extent of brain tumors. By evaluating the spatial distribution of substances correlated with metabolic activity in tumor tissue, including choline (Cho), N-acetylaspartate (NAA), creatine (Cr), lipids and/or lactate (LL), ^1^H-MRSI was used to differentiate different biological tumor components and to determine tumor borders for radiotherapy planning [74]. In a recent report, Grams et al. established and evaluated a multimodal imaging concept that included Cho/Cr ratio, NAA-concentration and rCBV derived from ^1^H-MRSI and DSC [55]. With this method, different groups of tumors with different multimodal imaging characteristics such as “true edema,” “cellular infiltration,” “cellular proliferation” and “tumor” were identified for patients with gliomas. Furthermore, the combination of normalized Cho, NAA, Cr and LL obtained by ^1^H-MRSI, ADC obtained by DWI and rCBV obtained by PWI was used to improve the delineation of GBMs [57], and the significant differences in multi-parameters (Cho, NAA, Cr, LL, ADC, and rCBV) in tumor mass and perienhancing areas could help in distinguishing among different tissues, not only among infiltrating tumor, vasogenic edema and normal tissue but also between high- and low-grade gliomas [56]. In another report, with the multimodal imaging data of DWI, PWI, and ^1^H-MRSI, the authors showed that a more optimal and safe planning target volume could be obtained [57].

For routine clinical application, opportunities and challenges exist side by side for the integration of multimodal MRI data into radiotherapy treatment planning. Because the multimodal imaging course takes a long time, which can induce motion artifacts by patients, and because of the instinct for registration errors to exist in the post-processing course due to the differences among imaging mechanisms, the accuracy of the definition of tumor borders might be affected. When strict tumor volume delineation is required due to the existence of some critical and sensitive anatomic structures around the tumor, the reduction of mismatches among multimodal images should be the most urgent issue to be resolved in MR multi-parameter analysis. Additionally, there are registration errors between localized ROI placement on MR images and the stereotactic biopsy locations, which can lead to uncertainty in the validation of those multimodality methods. To compensate for these registration errors, Hu et al. [64] used small craniotomy sizes and visually validated stereotactic image locations with intracranial neuroanatomical landmarks to minimize brain shift, leading to misregistration no greater than 1–2 mm.

### Future perspectives

These prospective studies mentioned above have proved that diffusion and perfusion MRI techniques and the quantitative metrics derived from these data are valuable for providing additional information about brain tumor pathophysiology. Patients with varied brain tumors can benefit considerably from the combination of diffusion and hemodynamic data with conventional MR examinations for tumor volume and invasive extent definition, as well as tumor response to treatment assessment in radiotherapy.

For the clinical implementation of diffusion and perfusion MRI data in radiotherapy, more effort are needed (1) to improve the imaging technology to shorten the MRI data acquisition time and reduce the motion artifacts of patients while retaining a relatively high image quality at the same time; (2) to investigate specialized and robust image registration algorithms for conventional and functional advanced MRI images to minimize mismatch errors in multimodal images post-processing; (3) to apply sufficient clinical experiments and data evaluations for comprehensive understanding and improvement of the correlation between detail histopathology results and functional information on MR parameter images and to prove the significance of functional MRI parameters; and (4) to determine the targets that should receive higher radiation doses based on the physiological information of functional MRI parameters correlated with tumor proliferation and aggressiveness.

Additionally, automatic tools or software integrated into a uniform radiotherapy treatment planning system (TPS) for rapid and reliable trimodal or multimodal image fusion of all of the functional imaging data are not yet available. The dual-modality image fusion techniques provided in current TPS can fuse and display two image sets in one panel and one operation, which is inconvenient for target definition in situations in which three or more sets of complementary information from conventional and functional MRI images are needed and used simultaneously. Thus, the development of new robust image fusion techniques that can fuse and display all image sets in one panel and one operation will be a major task in the coming years, and achieving this goal will also be helpful for conducting long-term prospective studies for the evaluation of the effectiveness of multimodal functional MRI techniques regarding the survival of patients with brain tumors.

## Conclusion

The combination of diffusion and hemodynamic data with conventional MR examinations for treatment planning and response assessment in radiotherapy will considerably benefit brain-tumor patients. Opportunities and challenges exist side by side for integration, and sufficient clinical experiments and data evaluations are needed. The development of new robust tools that can fuse and display all image sets into one panel and in one operation will be a major task in coming years, which could be helpful for conducting prospective studies for the evaluation of effectiveness.
